# Observation of Corneal Wound Healing and Angiogenesis Using Low-Vacuum Scanning Electron Microscopy

**DOI:** 10.1167/tvst.9.6.14

**Published:** 2020-05-16

**Authors:** Takeshi Arima, Masaaki Uchiyama, Akira Shimizu, Hiroshi Takahashi

**Affiliations:** 1 Department of Ophthalmology, Nippon Medical School, Tokyo, Japan; 2 Department of Analytic Human Pathology, Nippon Medical School, Tokyo, Japan

**Keywords:** low-vacuum scanning microscopy, corneal wound healing, neovascularization

## Abstract

**Purpose:**

Wound healing processes in a rat corneal alkali burn model were observed using low-vacuum scanning electron microscopy (LV-SEM), a new observation method that can use paraffin sections for light microscopic immunostaining.

**Methods:**

Injured cornea was observed under immunohistochemistry, LV-SEM, and transmission electron microscopy. In LV-SEM, periodic acid-methenamine silver staining was used to observe collagen and platinum blue staining was used to observe vascular endothelial cells. Analyses of the messenger RNA expression involved in neovascularization processes after wound creation were also performed.

**Results:**

LV-SEM depicted progression of corneal wound healing in a stereoscopic fashion. In neovascularization processes after wound creation, LV-SEM with osmification clearly demonstrated detachment of pericytes from the vascular endothelial cells, in association with up-regulation of angiopoietin-2 messenger RNA expression.

**Conclusions:**

LV-SEM enables high magnification observation of paraffin sections used for immunohistochemistry. LV-SEM provides easy, detailed observations and offers a promising new observational modality in the field of ophthalmology.

**Translational Relevance:**

High magnification analysis was easily available using LV-SEM with conventional paraffin sections for light microscopy.

## Introduction

Transmission electron microscopy (TEM) is a standard method for observing tissue ultrastructure,^1^^,^^2^ but installation of the requisite equipment is expensive and use requires specialized skills and time.^3^ A new technique, low-vacuum scanning electron microscopy (LV-SEM), can circumvent these drawbacks of TEM, and several recent studies have confirmed the usefulness of this method.^4^^–^^6^ LV-SEM provides high magnification images using formalin-fixed and paraffin-embedded tissue sections after simple and rapid processing,^7^ and is now widely used clinically and for research in various fields.^6^^,^^8^^–^^10^ One of the important advantages over TEM is that LV-SEM can be performed using conventional paraffin sections with standard staining for light microscopy, such as periodic acid-methenamine silver (PAM) or platinum blue (Pt) staining.^7^ PAM and Pt staining are performed to enhance the backscattered electron signal of biological materials.^11^ In addition, the area examined by light microscopy and immunofluorescence can be selectively evaluated in detail by LV-SEM.^7^ However, application of LV-SEM remains rare in the field of ophthalmology.^12^ The present study evaluated the usefulness of LV-SEM using a rat model of alkali burns. Furthermore, continuous changes in corneal neovascularization after alkali burn were observed using LV-SEM, and messenger RNA (mRNA) expression involved in the process was also analyzed.

## Methods

### Alkali Burn Model

Male Wistar rats (8 weeks old; Sankyo Laboratory Service, Tokyo, Japan) were used for all experiments in the present study (*n* = 5 at each time point). All animal experiments were conducted in compliance with the Experimental Animal Ethics Review Committee of Nippon Medical School, Tokyo, Japan, and all procedures conformed with the requirements of the Association for Research in Vision and Ophthalmic and Visual Research. A circular filter paper (diameter, 3.2 mm) that had been soaked in 1-N NaOH was placed on the central cornea in the right eye of each rat for 1 min while under general isoflurane anesthesia to create a corneal alkali burn. After a 1-min exposure, corneas were rinsed with 40 mL of physiologic saline. At each time point (6 h and 4, 7, and 14 days after alkali exposure), rats were euthanized by exsanguination under 3.5% isoflurane anesthesia. Enucleated eyes were used for immunohistochemical analysis, TEM, LV-SEM and real-time reverse transcription polymerase chain reaction (RT-PCR) after macroscopic examination. For RT-PCR analyses, dissected corneal tissues were immediately placed into RNA*later* solution (Life Technologies, Carlsbad, CA) and stored at –80°C. The contralateral uninjured normal rat cornea was used as a control sample.

### Histologic and Immunohistochemical Analysis

Eyes were enucleated and fixed in 10% neutral-buffered formalin and embedded in paraffin before observation under light microscopy. Subsequently, deparaffinized tissue sections (thickness, 2.5–10.0 µm) were used for immunostaining and LV-SEM. When using LV-SEM, deparaffinized tissues were stained with conventional PAM to identify collagens,^5^^,^^6^^,^^13^ and with Pt of the stock solution to identify vascular endothelial cells.^4^^,^^11^^,^^14^

We used the following primary antibodies for immunohistochemical analyses: (1) monoclonal mouse anti–α-smooth muscle actin (α-SMA; Dako, Glostrup, Denmark) to detect myofibroblasts and vascular pericytes^15^^,^^16^; and (2) monoclonal mouse anti-aminopeptidase P (JG12; Thermo Fisher Scientific, Rockford, MA) to detect vascular endothelial cells.^17^ Histofine Simple Stain rat MAX-PO (multi, Nichirei Bioscience, Tokyo, Japan) was used for secondary antibody in both immunostains. Regarding α-SMA–stained sections, osmification with 1% osmium tetroxide for 30 min after immunostaining was performed to enhance 3,3′-diaminobenzidine for LV-SEM observation.^6^

### TEM

Dissected corneas were cut into small pieces of about 1 mm × 2 mm, and fixed in 2.5% glutaraldehyde, post-fixed with 1% osmium tetroxide, and embedded in Epon 812 (Oken, Tokyo, Japan).^7^ Ultrathin sections were made with an ultramicrotome (Ultracut N, Reichert-Nissei, Tokyo, Japan) and stained with uranyl acetate and lead citrate.

### LV-SEM

In the present study, all types of sections for LV-SEM observation were embedded in paraffin. After PAM or Pt staining without a mounting cover glass, all sections were then immediately examined under LV-SEM (TM3030 tabletop microscope; Hitachi High-Technologies Corp., Tokyo, Japan).^4^^–^^6^ In observations of collagen, backscattered electron signal of collagen was enhanced by PAM staining and evaluated in the central area of the cornea. In observations of neovascularization in the corneal stroma, the contrast of the vascular endothelial cells were enhanced by Pt staining, while the contrast of the α-SMA–stained pericytes were enhanced by embedding osmium teroxide (1%). The duration of preparation for LV-SEM observation was completed within 1 day. Ultrastructural alterations of the corneal wound were assessed by LV-SEM using an acceleration voltage of 15 kV with 30 Pa for the backscattered electron detector.

### RT-PCR

In the present study, mRNA expression of angiopoietin (Ang)-1 and Ang-2 was examined as genes related to adhesion between pericytes and vascular endothelial cells during angiogenesis. Total RNA was extracted from the cornea using an RNeasy Mini Kit (Qiagen, Hilden, Germany) according to the protocol from the manufacturer. To ensure RNA concentration and purity (A_260_/A_280_), an ND-1000 v3.2.1 spectrophotometer (NanoDrop Technologies, Wilmington, DE) was used. Libraries of cDNA were created from 4 µg of total RNA using a High-Capacity cDNA Reverse Transcription kit (Applied Biosystems, Foster City, CA) in accordance with the protocol from the manufacturer. Gene expression levels were analyzed using 0.3 µL of cDNA with real-time detection of accumulated fluorescence in accordance with the manufacturer's manual (ABI PRISM 7900HT; Applied Biosystems). Normalized values for mRNA expression in each sample were calculated as the relative quantity of the housekeeping gene, β-actin. Primers used for real-time RT-PCR included: mβ-actin, 5′-ACC ACC ATG TAC CCA GGC ATT-3′ (forward) and 5′-CCA CAC AGA GTA CTT GCG CTC A-3′ (reverse); mAng-1, 5′-CAC CGT GAG GAT GGA AGC CTA-3′ (forward) and 5′-TTC CCA AGC CAA TAT TCA CCA GA-3′ (reverse); and mAng-2, 5′-CTT CAG GTG CTG GTG TCC A-3′ (forward) and 5′-GTC ACA GTA GGC CTT GAC CTC-3′ (reverse). SDS v2.3 software (Applied Biosystems) was used to perform all quantifications.

### Statistical Analyses

All results are expressed as mean ± standard deviation. Statistical analyses were performed using Mann-Whitney *U* test in Excel analytical software (Microsoft, Redmond, WA). *P* values of less than 0.05 were considered statistically significant.

## Results

### Observation of Normal Corneas Using LV-SEM

**Figure 1.**
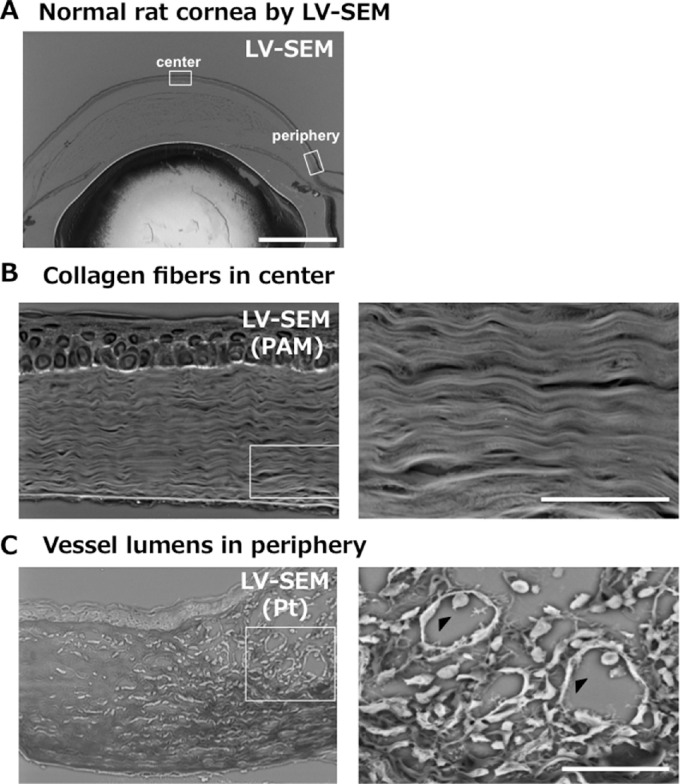
Observation of normal rat cornea by LV-SEM was easily and rapidly performed. (A) Wide-view LV-SEM enables designation of the observation point. In the present study, more detail images of the corneal center and periphery were observed. *Bar*: 1 mm. (B) LV-SEM with PAM staining enables capture of stereoscopic images of the corneal stroma. Increased magnification images of the boxed area shows the detailed pattern of collagen. *Bar*: 30 µm. (C) LV-SEM with Pt staining provides clear images of normal vessel lumens. Increased magnification images of boxed area shows vascular endothelial cells (*black arrowhead*) specified by Pt staining. *Bar*: 20 µm.

We first examined the structure of the normal cornea using LV-SEM. Figure 1A shows whole images of the eye under LV-SEM. LV-SEM enabled selective observation of the identified area, which is different from any other modality. All procedures for LV-SEM were completed in 1 day. LV-SEM provided high-powered fields (3000× magnification) and two-dimensional images from the slides of paraffin sections. Collagen fibers were clearly shown with PAM staining (Fig. 1B), whereas vascular endothelial cells were revealed by Pt staining (Fig. 1C).

### Comparison Between LV-SEM and TEM

**Figure 2.**
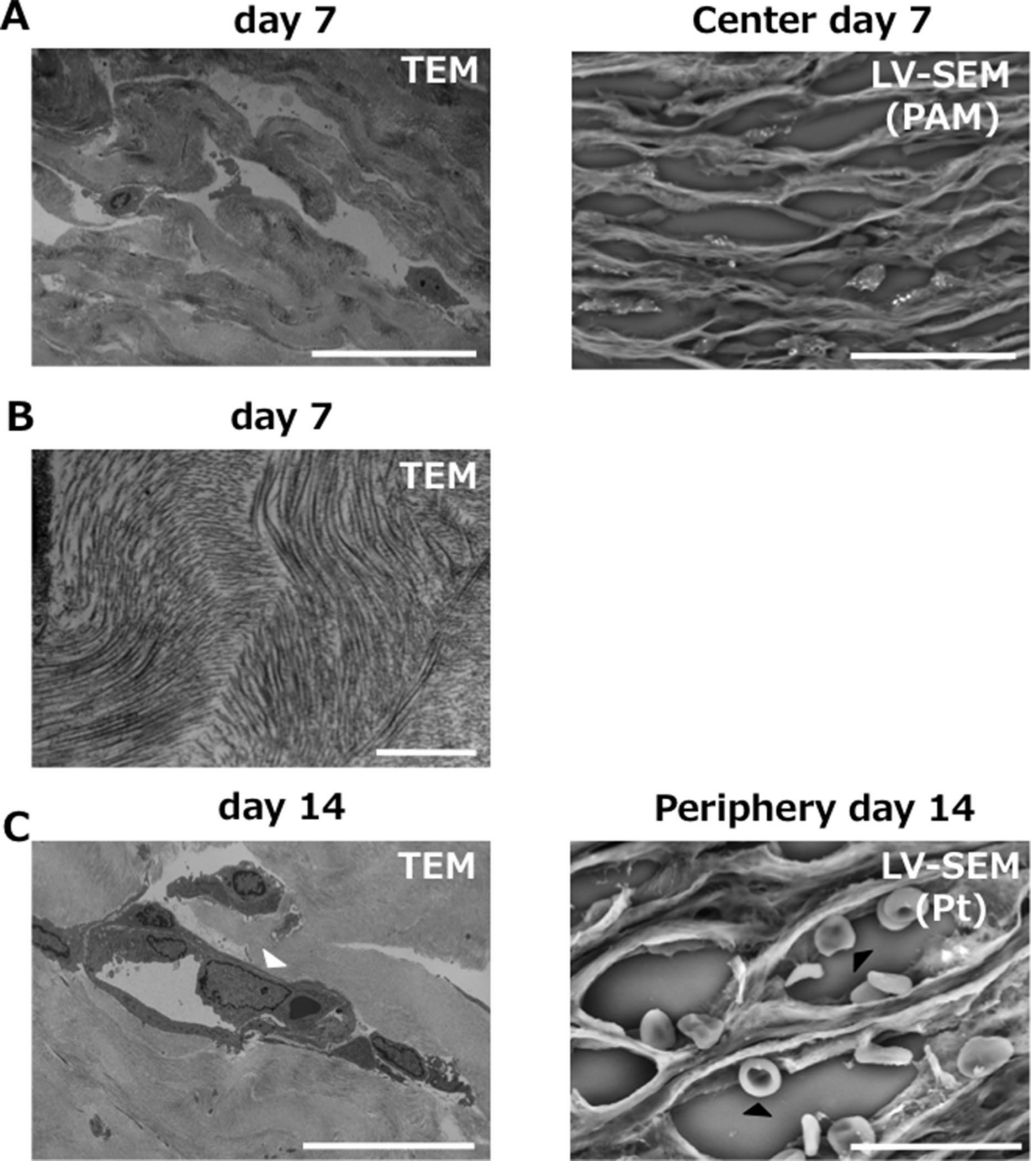
Collagen fibers and neovascularization were compared between TEM and LV-SEM using alkali-injured rat cornea. (A) The pattern of collagen fibers was disturbed and edema was observed between collagen fibers. LV-SEM showed more stereoscopic images than TEM. *Bar*: 30 µm. (B) Conversely, TEM provided more detailed images than LV-SEM. Collagen fibers not seen on LV-SEM were clearly observed. *Bar*: 1 µm. (C) Neovascularization was observed in both TEM and LV-SEM. TEM shows details of the nucleus (*white arrowhead*), whereas LV-SEM shows stereoscopic structure of red blood cells (*black arrowhead*). *Bar*: 20 µm.

Next, we examined structural alterations in corneal wounds after alkali injury. Morphologic changes to collagen in the corneal stroma were examined by TEM and LV-SEM (Fig. 2A). Although both TEM and LV-SEM clearly captured disturbances of collagen fibers and edema, LV-SEM provided stereoscopic images. Conversely, ultrahigh magnification LV-SEM was inferior to TEM, with which magnification of more than 100,000-fold is possible. The magnification achievable with LV-SEM is up to 10,000-fold. Figure 2B shows an image (30,000× magnification) of collagen fibers provided by TEM, which cannot be provided by LV-SEM. In the observation of neovascularization (Fig. 2C), both TEM and LV-SEM revealed that neovascularization involved vascular endothelial cells. The image of red blood cells clearly shows the difference between the two methods, with LV-SEM providing a scanning image of the object. The area was examined by light microscopy so that selectable search of observation area was possible in LV-SEM.

### Application of LV-SEM to Double Enhanced Microscopy

**Figure 3.**
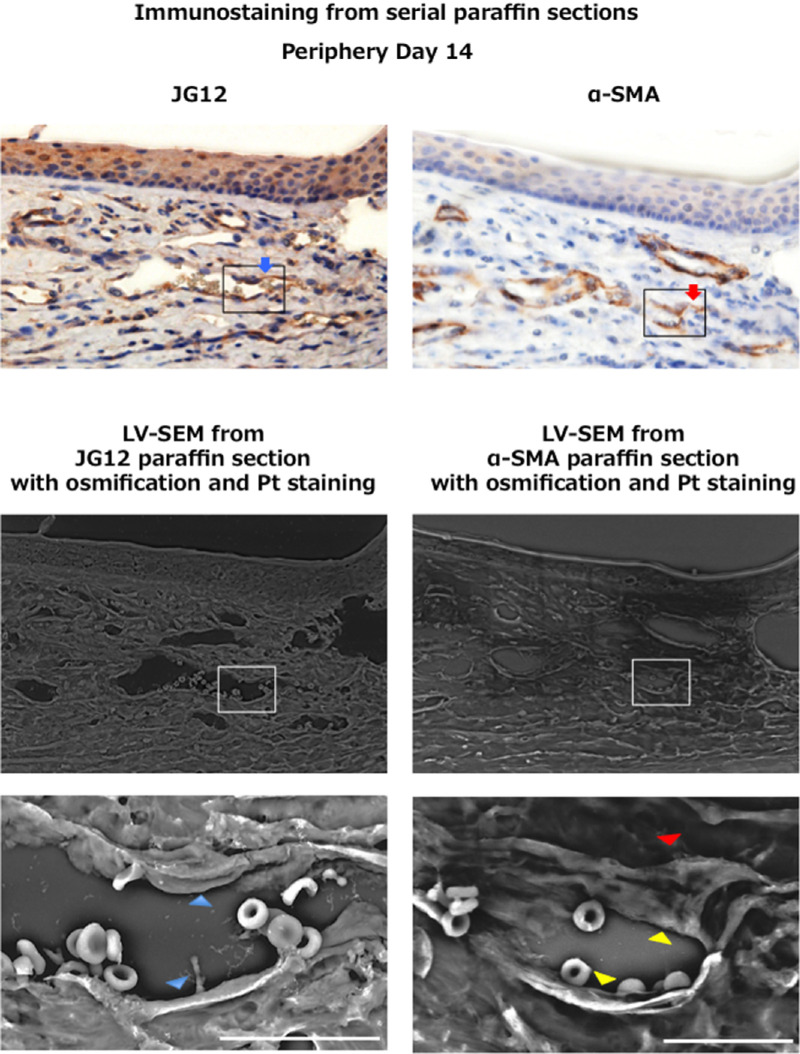
Double enhanced microscopy can be easily performed using LV-SEM of serial paraffin sections of alkali-injured cornea. Serial immunostaining shows that α-SMA stained pericytes (*red arrow*) seem separate from JG12-stained vascular endothelial cells (*blue arrow*). Because this method alone was uncertain, the same section with α-SMA immunostaining and osmification was subsequently examined under LV-SEM, with vascular endothelial cells revealed using Pt staining. LV-SEM enabled observation of stereoscopic electron micrographs at the same point as the immunostaining. Increased magnification images of the boxed area are also shown. Immunostained LV-SEM with Pt staining enables clear observation of vessel lumens. Vascular endothelial cells (*yellow arrowhead*) and α-SMA–stained pericytes (*red arrowhead*) seem to be dissociated. Because only vascular endothelial cells (*blue arrowhead*) were enhanced, LV-SEM from JG12 paraffin section with osmification and Pt staining could not distinguish vascular endothelial cells and pericytes. *Bar*: 20 µm.

One of the advantages of LV-SEM is that paraffin sections that have been prepared for immunostaining can be used. The region of interest for immunostaining can be observed by LV-SEM. Figure 3 shows images of immunostaining from serial paraffin sections of α-SMA–stained pericytes and JG12-stained vascular endothelial cells and images from LV-SEM of identical sections with α-SMA immunostaining. An image with increased magnification by LV-SEM of the same section is also shown. We performed osmification after α-SMA staining before LV-SEM, so that not only Pt-stained vascular endothelial cells, but also pericytes were enhanced in LV-SEM. Double enhanced microscopy was therefore possible by applying immunostained sections for LV-SEM.

### Observation of Consecutive Changes in Neovascularization Using LV-SEM

**Figure 4.**
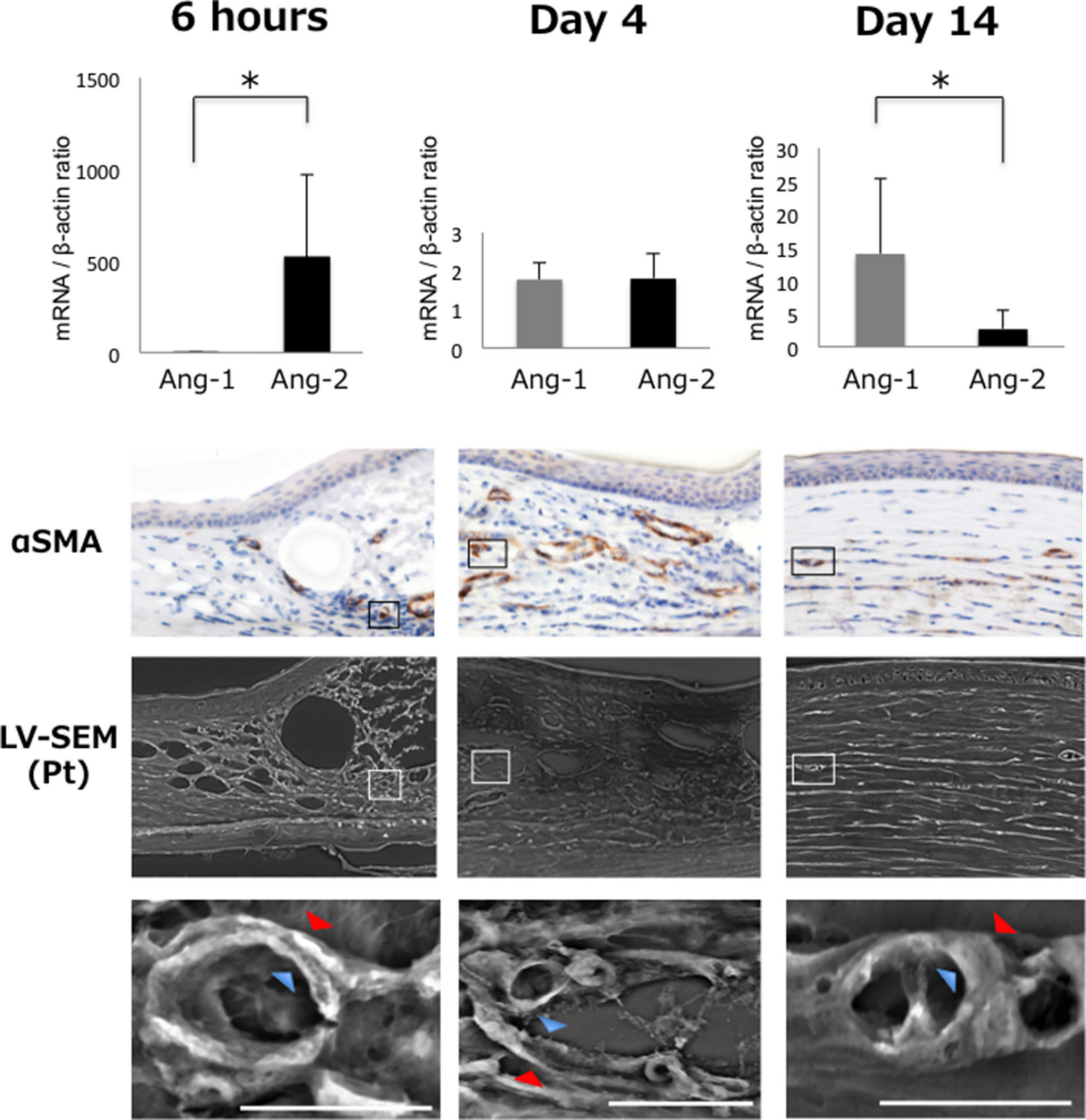
Double enhanced microscopy by LV-SEM with PCR assay allowed detailed observation of consecutive changes to neovascularization. The position of binding between pericytes and vascular endothelial cells after changes in the mRNA expression of Ang-1 and Ang-2 was investigated over time. At 6 h after alkali injury, mRNA expression of Ang-2 was markedly higher than that of Ang-1. From day 4 to day 14, the mRNA balance of Ang-1 and Ang-2 was reversed, and mRNA expression of Ang-1 increased significantly. Reflecting these results, LV-SEM images showed pericytes (*red arrow*) enhanced by osmification were beginning to separate from Pt-stained vascular endothelial cells (*blue arrow*) from 6 h to day 4, and reattachment was seen on day 14. * *P* *<* 0.05. *Bar*: 10 µm.

Next, we observed consecutive changes associated with corneal neovascularization after injury under double enhanced microscopy using LV-SEM (Fig. 4). At 6 h after alkali injury, Ang-2 mRNA expression was markedly increased. On day 4, pericytes detached from the vascular endothelial cells, and red blood cells were observed in gaps. However, on day 14, vascular endothelial cells and pericytes adhered again accompanying reverse in the mRNA balance of Ang-1 and Ang-2. These changes were clearly observed two-dimensionally by LV-SEM to visualize the developmental course of neovascularization.

## Discussion

The results of this study demonstrated the usefulness of LV-SEM, which provided detailed stereoscopic images of the tissue easily and rapidly compared to TEM. The experiment time of TEM need about 2 weeks, whereas that of LV-SEM was within 1 day. LV-SEM showed cross-sectional ultrastructural changes to collagen, with precision similar to that from TEM. The greatest advantage of LV-SEM is that conventional paraffin sections for light microscopy can be used so that ultramicroscopic images can be obtained in the same sections for immunostaining.^4^^–^^7^^,^^9^^,^^18^ This advantage enables to search selectively the area to observe in LV-SEM, which can not be done in TEM. Slides that have been used for light microscopy can be used for LV-SEM so that retrospective analysis of two-dimensional structures is possible.^5^ Another important advantage is that LV-SEM in combination with heavy metal staining can reveal detailed structures using bright/dark contrast.^5^ In the present study, collagen fibers and basement membranes were stained by PAM, whereas vascular endothelial cells and pericytes were stained by Pt. Staining intensity of antigen-specific 3,3′-diaminobenzidine was enhanced using the osmium black method.^6^ Consequently, contrast of the two tissues distinguished by immunostaining and heavy metal staining was useful for double enhanced microscopic observation. Regarding observation of ultrafine structures, however, TEM is superior to LV-SEM. A comparison between LV-SEM and TEM is shown in Supplementary Figures S1 and S2.

We then applied double enhanced microscopy by LV-SEM to observe the process of neovascularization after alkali burn. We also performed real-time RT-PCR for mRNA expression of vascular endothelial growth factor, Ang-1, and Ang-2, as well-known factors involved in neovascularization.^12^^,^^19^^–^^22^ In normal vascular walls, pericytes play roles in suppressing endothelial cell differentiation and proliferation. Ang-1 augments adhesion of pericytes to endothelial cells and promotes vascular maturation,^23^ whereas Ang-2 promotes embrittlement of mature blood vessels, which includes detachment of pericytes from endothelial cells and enhancement of reactivity to vascular endothelial growth factor.^24^ Ang-2 thus initiates endothelial cell proliferation by dissociating pericytes and vascular endothelial cells. Our previous study observed the process of corneal neovascularization and found that during the process, Ang-2 expression increased first, followed by vascular endothelial growth factor up-regulation.^12^ Agreeing with the previous report, detached pericytes and vascular endothelial cells were clearly observed in the present study using LV-SEM, and the presence of these cells was associated with increases in Ang-2. Adding osmification in LV-SEM enabled clarification of the distinction between pericytes and vascular endothelial cells, allowing clear demonstration of the separation of the two cell types. This approach may be useful in examining not only corneal neovascularization, but also retinal neovascularization.

In conclusion, we observed corneal wound healing processes in an alkali burn model using LV-SEM. The results suggest that LV-SEM offers advantages over TEM in terms of simplicity, rapidity, stereoscopic views, selectivity of viewpoints, and discriminant analysis. Although LV-SEM does not match TEM in the ability for qualitative diagnosis, this method may be useful for rapid diagnosis while awaiting more precise results.

## Supplementary Material

Supplement 1

Supplement 2
