# Supplementary material for: Observation of Corneal Wound Healing and Angiogenesis Using Low-Vacuum Scanning Electron Microscopy
Source: Transl Vis Sci Technol. 2020 May 16;9(6):14. doi: 10.1167/tvst.9.6.14 (PMC7408877; doi:10.1167/tvst.9.6.14)
Supplement: Supplement 1 [file tvst-9-6-14_s001.pdf]

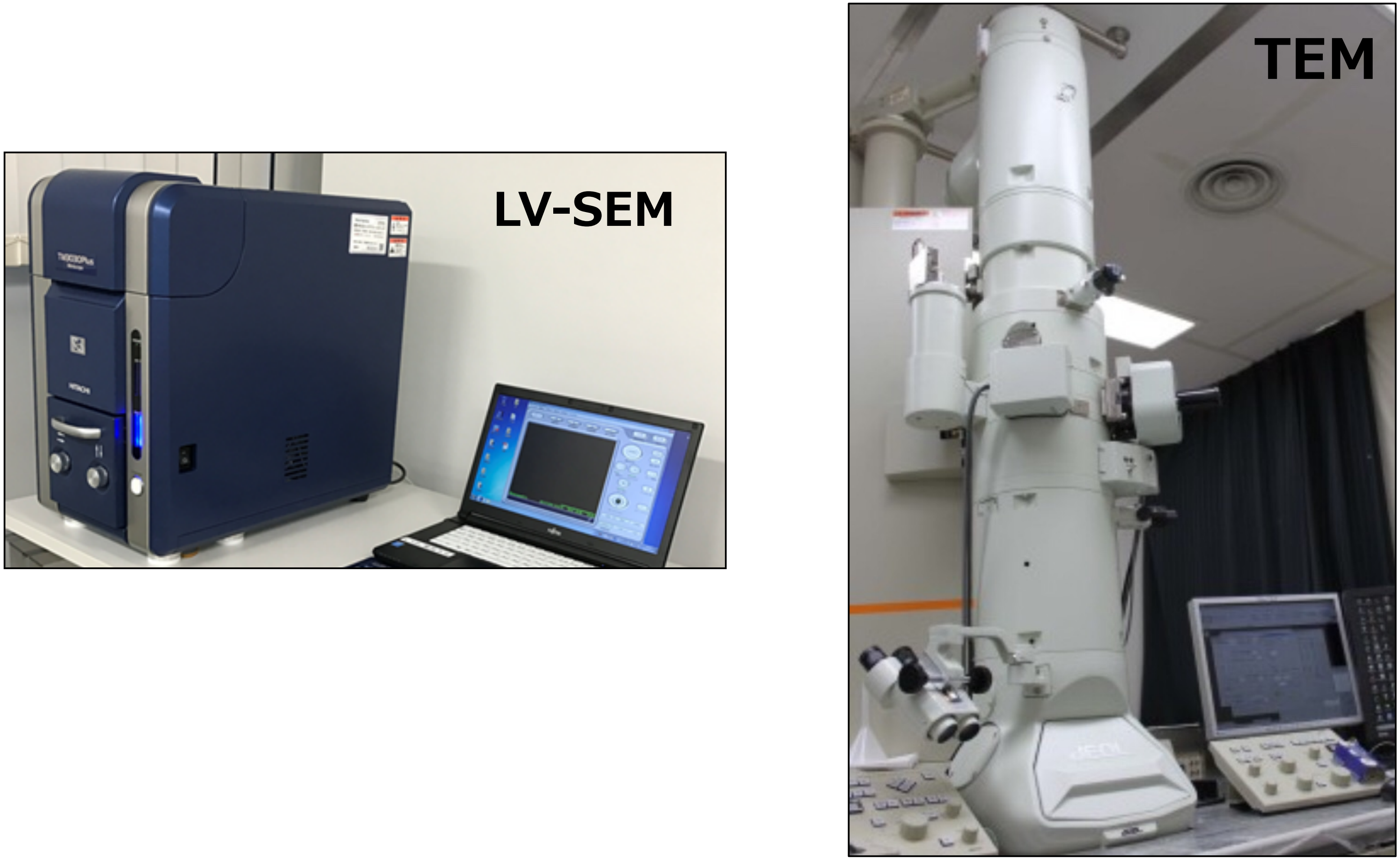

Supplementary Figure S1. Images of the LV-SEM and TEM systems. LV-SEM can be installed in a narrow space and does not require special equipment such as a TEM.
