# Supplementary material for: Observation of Corneal Wound Healing and Angiogenesis Using Low-Vacuum Scanning Electron Microscopy
Source: Transl Vis Sci Technol. 2020 May 16;9(6):14. doi: 10.1167/tvst.9.6.14 (PMC7408877; doi:10.1167/tvst.9.6.14)
Supplement: Supplement 2 [file tvst-9-6-14_s002.pdf]

	LV-SEM	TEM
Difficulty of method	Easy	Difficult
Experiment time	Within 1 day	About 1–2 weeks
Micrograph	Three-dimensional	Two-dimensional
Capital investment / Maintenance per year	About 45,000 US dollars / up to 9,000 US dollars	About 545,000 US dollars / about 18,000 US dollars
Area to be observed	Selectable from light microscopic slides	Unable to select
Magnification	Within 10,000-fold	Over 100,000-fold

Supplementary Figure S2. Comparison between LV-SEM and TEM. LV-SEM is superior to TEM in terms of convenience, cost, and selectivity of observation, but TEM can capture images at ultrahigh magnification, which cannot be provided using LV-SEM.
